# Cognitive Behavioral Therapy Treating Juvenile Fibromyalgia

**DOI:** 10.7759/cureus.12496

**Published:** 2021-01-05

**Authors:** Salim Habib, Emad U Alatassi, Anas Mahmoud, Mohamed Rafat Akkad Wattar, Mohamad Khaled Almujarkesh

**Affiliations:** 1 Internal Medicine, Almouassat University Hospital, Damascus, SYR; 2 Internal Medicine, University of Damascus, Damascus, SYR; 3 Internal Medicine, Al Noor Specialist Hospital, Makkah, SAU; 4 Internal Medicine, Icahn School of Medicine at Mount Sinai Queens, New York, USA; 5 Internal Medicine, Danylo Halytsky Lviv National Medical University, Lviv, UKR; 6 Internal Medicine/Gastroenterology, University of Damascus, Damascus, SYR

**Keywords:** fibromyalgia

## Abstract

Juvenile ﬁbromyalgia (JFM) syndrome is a condition under the spectrum of chronic pain syndrome. It is characterized by chronic musculoskeletal pain with multiple tender points and other associated symptoms. Diagnosis requires ruling out organic causes in addition to at least five out of 18 tender points and fulfilling three out of 10 criteria. JFM can be debilitating and overwhelming to both the patient and the physician. Management requires an incredibly careful multidisciplinary approach, including psychotherapy and physiotherapy among others.

## Introduction

Here, we report a 17-year-old girl with juvenile fibromyalgia (JFM) causing debilitating chronic pain that led to a severe disability requiring her to be wheelchair-bound for eight months. Multiple pain management protocols were not beneficial in either controlling her pain or relieving her disability. The patient's condition improved significantly by physical rehabilitation combined with cognitive behavioral therapy.

## Case presentation

A 17-year-old girl was referred to the rheumatology clinic complaining of pain, fatigue, and morning stiffness for the last few months that left her wheelchair-bound. She had been in her usual state of good medical health until about six months ago when she developed a runny nose and low-grade fever followed by right-sided lower motor neuron facial paralysis. She was treated with acyclovir, 500mg three times daily for 10 days, and prednisolone, 60mg for five days then tapered by 10mg per day for a total of 10 days, after which facial paralysis improved. Three weeks later, the patient started to have intolerable throbbing headaches and right facial pain. Her symptoms progressively deteriorated to become excruciating sharp pain in her lower extremities, exacerbated by physical and mental activities, which led her to quit school and be dependent on a wheelchair approximately eight weeks from the onset of the initial complaint. Further history did not show any previous psychological, past medical or family history.

On functional assessment, her pain, morning stiffness, and fatigue were assessed, and on patient global assessment, all were full 10 (where 1 is the least and 10 is the most severe). On physical examination, vital signs were within normal limits. She was tearful and in apparent distress. Musculoskeletal examination showed a full range of motion at all joints, but with subjective soft tissue swelling, pain, and hypersensitivity to touch in proximal and distal muscle groups of lower extremities with seven out of 18 tender points. On neurological examination, she was oriented to place, person, and time. Cranial nerves were grossly intact with no facial palsy and no gross motor deficit with 5/5 power throughout. There were some areas of hypersensitivity and tenderness all over but with no gross sensory loss. Finger-nose-finger and Romberg tests were normal, and reflexes were 2+ and symmetrical bilaterally. Gait could not be assessed as she refused to walk due to pain. Examination of other systems was unremarkable.

To rule out structural neurological and organic causes, the neurology team obtained brain and spinal MRI, which were normal. Lumbar puncture was performed with synovial fluid analysis and polymerase chain reaction (PCR), which came back negative. Laboratory evaluation including complete blood count (CBC), comprehensive metabolic panel (CMP), erythrocyte sedimentation rate (ESR), C-reactive protein (CRP), creatine phosphokinase (CPK), and thyroid function were all within normal limits. Antinuclear antibody, anti-Sjögren's syndrome-related antigen A (anti-SSA/Ro), and rheumatoid factor (RF) were also negative. The patient had three out of 10 (chronic headache, fatigue, and subjective soft tissue swelling) and over five out of 18 tender points for more than three months, consequently was diagnosed as JFM according to Yunus and Masi criteria [1].

Over the following few months, the patient was referred to multidisciplinary adolescent psychiatry where multiple pain treatments including trials of acetaminophen, non-steroidal anti-inflammatory drugs (NSAIDs), triptans, gabapentin, pregabalin, fluoxetine, nerve blocks, and Botox injections were unsuccessful, and the patient remained bound to a wheelchair and unable to ambulate. The turning point was when the patient started physical rehabilitation and cognitive behavioral therapy (CBT), where she responded very well, and all medications were discontinued. On her follow-up visit, she was pain-free, able to walk unassisted, and went back to school and social life as she recovered almost completely after over eight months of disability.

## Discussion

**Figure 1:**
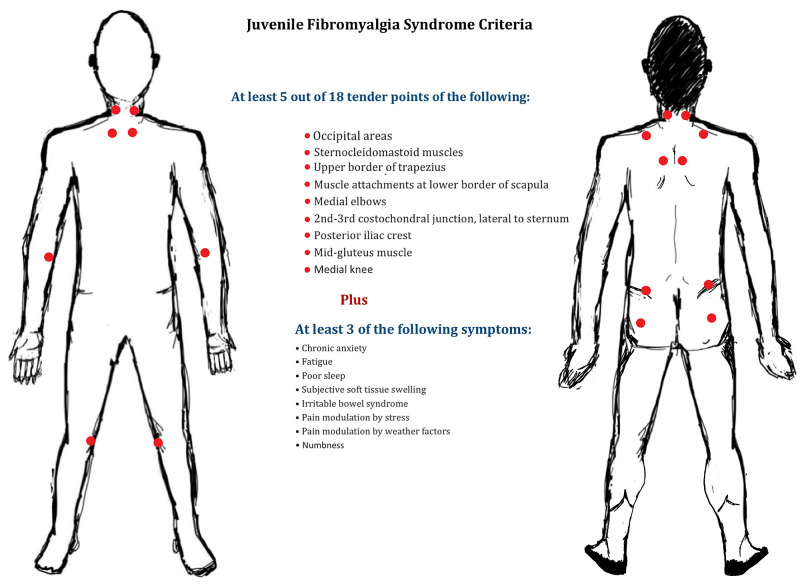
Juvenile Fibromyalgia Syndrome Diagnostic Criteria Adapted from Yunus and Masi [1].

JFM is a chronic musculoskeletal non-inflammatory pain syndrome. Although it does not lead to permanent joint or muscle damage, it can, however, increase avoidant behavior such as lowering physical activity, which paradoxically increases the pain and causes significant impairment in the patient’s life leading to physical disability [2]. It is estimated to affect up to 35% of children, especially female adolescents, and may continue into adult life, as seen in 17% of adult fibromyalgia cases [3,4]. The characteristic presentation is a diffuse musculoskeletal pain with multiple pathognomic tender points on palpation associated with other symptoms such as fatigue, chronic headaches, irritable bowel syndrome, and subjective soft tissue swelling that might be accompanied by depression, anxiety, or poor sleep. The pathophysiological mechanism of pain in fibromyalgia can be regarded as a pain hypersensitivity disease caused by abnormal pain transmission and/or pain perception [5]. JFM was first described by Yunus and Masi in 1985, and they proposed diagnostic criteria which are a widespread pain in more than three areas, for over three months, along with five out of 18 tender points, after ruling out organic and infectious etiologies (Figure 1) [1].

Recommended management of JFM comprises a multidisciplinary approach requiring psychological support, regular exercise, and physical therapy, along with pharmacological treatment. Psychological intervention, especially CBT, even without the aid of drugs, is an effective treatment for chronic pain conditions as it significantly reduces functional disability and relieves the other symptoms [6,7]. Exercise and physical therapy have been proved effective; however, the patient should be advised that some mild increase in fatigue and pain is expected at the beginning. Pharmacological treatment alone is unlikely to cause improvement in JFM. Multiple studies have shown the superiority of behavioral treatment over medical treatment, which is well proved in our case. However, it promotes participation in the aforementioned treatments as it decreases pain, improves sleep, and relieves anxiety [8,9]. Medications used usually fall in the analgesics, antidepressants, and anticonvulsants categories.

## Conclusions

JFM is a chronic condition with an unknown etiology. It can severely diminish the daily activity and productivity of the patient and family. We highlight the importance of a multidisciplinary approach and non-pharmacologic treatment, which can significantly reduce disability and improve the patient's quality of life.
